# Examination of the functions and mechanism of *KCP* in mediating paclitaxel resistance in cervical squamous carcinoma cells

**DOI:** 10.3389/fonc.2025.1550032

**Published:** 2025-04-14

**Authors:** Yue He, Jian-Qing Xu, Jing-Jing Zhang, Zhen-You Liu, Chen Ji, Yang Liu, Yun-Fan Wang, Ming Wang, Yu-Mei Wu, Yan Wang

**Affiliations:** Department of Gynecological Oncology, Beijing Obstetrics and Gynecology Hospital, Capital Medical University, Beijing Maternal and Child Health Care Hospital, Beijing, China

**Keywords:** Kielin/chordin-like protein, paclitaxel resistance, cervical squamous cell carcinoma, mechanism, functions

## Abstract

**Objective:**

To evaluate the mechanism of Kielin/chordin-like protein (*KCP*) in the resistance of cervical cancer cells to paclitaxel.

**Method:**

A cervical squamous carcinoma cell line (SiHa) with *KCP* knockout was constructed and treated with paclitaxel. Key cell functions were assessed by colony formation assay, measurement of cell proliferation by 3-(4,5-dimethylthiazol-2-yl)-2,5-diphenyltetrazolium bromide (MTT) assay, and FACS-based detection of apoptosis. The downstream mechanism of *KCP*-mediated resistance to paclitaxel was then examined using human gene chip detection and IPA bioinformatics analysis, and qPCR analysis was used to validate its downstream genes.

**Results:**

①Functional studies of SiHa cells showed that *KCP* knockout (sgRNA) inhibited colony formation and proliferation of SiHa cells in the presence of paclitaxel (*p*<0.05). ②Using a whole human genome microarray, a total of 491 differentially expressed genes were identified in *KCP* knockout versus the NC SiHa cells. IPA-based bioinformatics analysis of upstream regulators showed that SPI1 was strongly activated and that SPI1 inhibited *CCND1* and activated *PML* and *CEBPA*, which is consistent with results from gene chip analysis showing *CCND1*, *PML*, and *CEBPA* expression after *KCP* knockout. ③A total of 30 differentially expressed genes associated with tumor cell proliferation were identified by gene microarray and IPA analyses. The changes in the aforementioned genes after *KCP* knockout were verified by qPCR, and *SERPINB3* and *CEBPA* expression were significantly lower and higher, respectively, compared to in the control group.

**Conclusion:**

*KCP* increased resistance of cervical cancer to paclitaxel by enhancing cell proliferation and colony formation. We observed that *KCP* could act positively on the downstream gene *SERPINB3* and negatively on the downstream gene *CEBPA* to affect the resistance of cervical carcinoma cells to paclitaxel.

## Highlights

- *KCP* is associated with paclitaxel chemoresistance in cervical cancer.
- *KCP* can act as an upstream gene via SPI1 on downstream genes to participate in the regulatory network of replication of murine herpesvirus 4 and replication of vesicular stomatitis virus.
- *KCP* can also act positively on the downstream gene *SERPINB3* and negatively on the downstream gene *CEBPA* to participate in the proliferative function of cervical squamous carcinoma cells and affect the resistance of cervical carcinoma to paclitaxel.

## 1 Introduction

Cervical cancer is the fourth most common malignancy affecting women worldwide. Approximately 90% of diagnosed cases are squamous cervical cancer (1). According to the data published by the International Agency for Research on Cancer (IARC) in 2020, approximately 604,000 new cervical cancer cases and 342,000 deaths were reported globally (2). China has the highest number of new cases and deaths (3), with 131,500 new cervical cancer cases and 53,000 deaths each year, accounting for 18.4% of female cancer-related mortality in China (4). In developing countries, the 5-year survival rate for patients with cervical cancer is < 50%. For patients diagnosed in stages III or IV, the 5-year survival rate is only 5% to 15%, thus posing a serious threat to the lives of women (2).

Paclitaxel, which is a first-line chemotherapy drug for cervical cancer, is widely used in patients with intermediate to advanced squamous cell cervical cancer and plays an important role in the chemotherapy of cervical squamous cell carcinoma. However, paclitaxel resistance is the main cause of treatment failure, and overcoming paclitaxel resistance has become the most important clinical problem for gynecological oncologists.

In 2002, Abreu’s research group (5) discovered that Kielin/chondrin like protein (KCP) can affect the progression of certain chronic and metabolic diseases by regulating extracellular signals. KCP is a secreted protein that is expressed at higher levels in embryonic germ and kidney tissues, and at lower levels in normal adult tissues. In the TCGA database, it was found that KCP is highly expressed in various tumor tissues, including bladder ureteral cancer, gallbladder cancer, colon cancer, head and neck tumors, esophageal cancer, and renal cancer (http://starbase.sysu.edu.cn/panGeneDiffExp.Php). KCP is also significantly highly expressed in cervical cancer, especially in young Asian squamous cell carcinoma patients, and affects prognosis. We previously successfully constructed a Kielin/chordin-like protein (*KCP*) knockout model of cervical squamous cell carcinoma cells *in vitro* using CRISPR/Cas9 gene editing technology, we designed a single lentiviral vector to deliver Cas9 sgRNA and a fluorescence selection marker to the target cells (6). SiHa is a cervical squamous cell carcinoma line containing human papillomavirus that is resistant to cisplatin. In this study, we preliminarily confirmed the regulatory function of *KCP* in mediating paclitaxel resistance of SiHa cells via cell function assays, whole human genome microarrays, and Information Process Analysis (IPA) bioinformatics analysis. This study is the first to explore the role of *KCP* in paclitaxel resistance in cervical cancer.

## 2 Materials and method

### 2.1 Materials

The human cervical squamous carcinoma cell line (SiHa) was purchased from the cell bank of the Chinese Academy of Sciences (Shanghai, China). The following materials were used: DMEM medium (HyClone, 130701), fetal bovine serum (Hangzhou Sijiqing Company, VS500T), trypsin (GIBCO, 25200-072), paclitaxel (PTX) (PTX, Beijing Xiehe Pharmaceuticals, 141101), *KCP*-overexpressing lentivirus, *KCP* gene sgRNA lentivirus (Shanghai Genechem Co.,Ltd), MTT (Shanghai Dingguo Biotechnology Co., Ltd., DH343-2), apoptosis kit (eBioscience, 88-8007), human gene expression profiling chip GeneChip^®^ PrimeView^®^ human (Affymetrix, 902487), GeneChip^®^ Hybridization Wash and Stain Kit (Shanghai GeneChem Co., Ltd), Agilent RNA 6000 Nano Kit (Agilent), TRIzol™ (Shanghai Pu Fei Company, 3101-100), M-mlV (Promega, M1705), dNTPs (Promega, U1240), RNase Inhibitor (Promega, N2115), oligo dT (Shanghai Biotech Company, B0205), Bulge-Loop™ miRNA qPCR Primer Set (Guangzhou Ruibo Company), KCP primary antibodies (Abcam, AB139956), β-actin (Abclonal, RP02968LQ), anti-rabbit secondary antibody (ZSGB-Bio, ZB2301) and SYBR^®^ Green Master Mixture (TAKARA, DRR041B).

### 2.2 Equipment

The following instruments were used: fluorescence microscope (Olympus, IX71), CO_2_ incubator (Sanyo, MCO-15A), inverted microscope (Shanghai Cai Kang Optical Instruments Co., Ltd., XDS-100), centrifuge [Thermo Fisher Scientific (China) Co., Ltd., Fresco 21], biological safety cabinet (Shanghai Zhen Yang Chuang Air Purification Equipment Co., Bio-1200-II-A2), SDS-PAGE protein electrophoresis instrument (Shanghai Tianneng Company, VE-180), protein electrophoretic transfer instrument (Shanghai Tianneng Company, VE-186), high-speed refrigerated centrifuge (Thermo, Fresco 21), flow cytometer (Millipore, C6 PLUS), NanoDrop™ 2000 (Thermo), cellometer (Nexcelom Cellometer), 2100 Bioanalyzer (Agilent, Auto 1000), NanoDrop™ spectrophotometer (Thermo, 2000/2000C), GeneChip^®^ Hybridization Oven 645 (Thermo, 00-0331), GeneChip^®^ Fluidics Station 450 (Thermo, 00-0079), GeneChip^®^ Scanner 3000 (Shanghai GeneChem Co., Ltd, Thermo 00-0362), stabilised flow electrophoresis instrument (Shanghai Tianneng Company, EPS-600), qPCR instrument (Roche, LightCycler^®^ 480 II), and a microplate reader (Tecan infinite, M2009PR).

### 2.3 Methods

#### 2.3.1 Cell culture

SiHa cells infected with KCP sgRNA lentivirus (sgRNA group; sgRNA sequence: GGACTCACCTTCCGCG- CCCG) and the control lentivirus (normal cells group, NC group; control lentivirussequence: CGCTTCCGCGGCCCGTTCAA) were incubated in DMEM medium containing 10% fetal bovine serum at 37°C in a 5% CO_2_ incubator. The medium was replaced every 2 to 3 day, and the cells in the logarithmic growth phase were extracted for the experiments. We applied Western Blot (WB) to verify whether KCP sgRNA was successfully transfected.

#### 2.3.2 PTX concentrations for experiments

We determined the IC50 and concentration of PTX in earlier studies. PTX concentrations of 2 to 4 ng/ml inhibited cell growth by 23.34% to 74.12%, respectively (7), 2-4ng/ml can be chosen as the concentration for cell function experiments. After multiple repeated experimental evaluations, we finally chose 2ng/ml for colony formation assay experiments, 4ng/ml for FACS-based detection of apoptosis and MTT experiments.

#### 2.3.3 Western blot

Approximately 1×10^6^ siha cells were collected by centrifugation, and ripa lysate was added to lysis to release proteins. Protein concentration was determined using bicinchoninic acid (Pierce, Waltham, MA, USA). A protein sample of 3 ug/uL was prepared. Electrophoresis was carried out in 4% stacking gel at 90 V for 20 minutes and 8% separating gel at 120 V for 1 hour, then the proteins were transferred to a polyvinylidene fluoride membrane at 380 A for 90 minutes. The membrane was blocked in 5% non-fat milk for 1 hour at 25°C and incubated with primary antibodies (KCP antibody, Abcam, AB139956) at a ratio of 1:1,000 at 4°Covernight. Next, the membrane was incubated with secondary antibodies (anti-rabbit secondary antibody; Proteintech) at a ratio of 1:10,000 for 1 hour at 25°Cand protein bands were visualized using an Amersham Imager 600.

#### 2.3.4 Colony formation assay

SiHa cells from the sgRNA and NC groups were harvested by trypsinization and counted, and the cell suspension was diluted before being inoculated in six-well plates at a density of 500 cells per well. The plates were then incubated in a cell incubator for 14 d until visible cell colonies appeared. On day 3, paclitaxel (2ng/ml) was added according to the experimental design, and no treatment was administered to the no-drug group. The medium was then discarded, and the cells were fixed in 4% paraformaldehyde for 15 min, followed by removal of the fixative. A total of 1 ml of crystal violet was added, and staining was performed for 10 min. The crystal violet was slowly washed away with running water. The colonies were counted directly with the naked eye or under a microscope for cell counts of more than 50, and the colony formation rate was calculated as follows: colony formation rate = (number of colonies/number of cells inoculated) × 100%.

#### 2.3.5 Cell proliferation assay based on 3-(4,5-dimethylthiazol-2-yl)-2,5-diphenyltetrazolium bromide

SiHa cells derived from the sgRNA and NC groups were trypsinized and counted, and the cell density was adjusted to 2×10^3^ cells/ml, followed by inoculation in 96-well plates at a volume of 100 ul/well. On day 2, paclitaxel (4ng/ml) was added according to the experimental design, and no treatment was administered to the no-drug group. The cells were continually incubated for a total of 5 days, after which a solution containing 20 ul of MTT (5mg/ml) was added to each well, followed by incubation for four hours. The culture medium was completely aspirated (care was taken not to aspirate the formazan granules at the bottom of the wells), and 100 ul of DMSO was added to each well to dissolve the formazan granules. After shaking with an oscillator for 3 to 5 min, the OD value was measured at 490 nm using a microplate reader. The relative viability of the cells was calculated from the OD values, and cell growth curves were generated. OD490/fold was the OD490 increase relative to day 1 for each experimental group from day 1 to day 6, representing the proliferation increase for each day. The inhibition rate was [1 - OD490/fold (sg*KCP* + paclitaxel)/OD490/fold(sg*KCP*)] × 100%.

#### 2.3.6 FACS-based detection of apoptosis

Drug-induced apoptosis was assayed when the cells in six-well plates of each experimental group exhibited approximately 70% confluence (on day 7 after lentivirus infection). On day 4, paclitaxel (4ng/ml) was added according to the experimental design, and no treatment was administered to the no-drug group. The cells in the supernatant were collected and added to the adherent cell fraction. After trypsin digestion of the adherent cells, the complete medium was resuspended into a cell suspension and added to the supernatant cells in the same centrifuge tube (5ml), with three replicate wells per group (cell number≥5×10^5^/process). They were then centrifuged at 1,300 rpm for 5 min, and the supernatant was discarded. The cell pellet was washed with pre-cooled D-Hanks buffer (pH=7.2~7.4) at 4°C. The cell pellet was washed once with 1× binding buffer, centrifuged at 1,300 rpm for 3 min, and then resuspended in a total of 200 ul of 1× binding buffer. A total of 10 ul of Annexin V-APC was added to the buffer for staining, and the solution was allowed to stain for 10–15 min at room temperature while protected from light. A total of 400 to 800 ul of 1×binding buffer was added depending on the number of cells, and the flow cytometry assay was performed with 1000 cells per run.

#### 2.3.7 Gene chip detection and ingenuity pathway analysis bioinformatics analysis

Total RNA was extracted using the TRIzol™, and the samples were inspected using NanoDrop™ 2000 and Agilent Bioanalyzer 2100 instruments. Qualified samples were subjected to gene microarray testing according to the 3’ IVT PLUS kit instructions (Affymetrix item no. 902487). Based on the expression of differential genes detected by the gene microarray, we use the stats package in R language software (stats and graphics packages) to count the number of chip probe sets and plot them, IPA bioinformatics analysis was performed to predict the interaction relationship between the relevant detected genes and the target gene encoding *KCP* and to map the gene network (8, 9). Differentially expressed genes were defined as the FDR>1.3 or <-1.3, p-value<0.05.(URL:https://digitalinsights.qiagen.com/products-overview/discovery-insights-portfolio/analysis-and-visualization/qiagen-ipa/).

#### 2.3.8 q-PCR validation of downstream genes

Cells were collected (six-well plate, 80% cell density) and centrifuged at 2,000 rpm for 5 min. A total of 1 ml of TRIzol™ was added to the cell pellet and mixed thoroughly before allowing the solution to stand for 5 min at room temperature. The solution was then transferred to a new EP tube with a volume of 1.5 ml. A total of 200 ul of chloroform was added to each tube, which was turned upside down by hand before letting it stand for 8 min at room temperature. The solutions were then centrifuged at 12,800 rpm for 15 min at 4°C. The upper layer was transferred to a new EP tube with a volume of 1.5 ml. An equal volume of pre-cooled isopropanol was added, and the solution was mixed well before allowing it to stand for 10 min at 4°C. After centrifugation for 12 min at 12,800 rpm at 4°C, the supernatant was discarded, and the precipitate was washed with 1 ml of 75% ethanol. It was then centrifuged for 5 min at 11,800 rpm at 4°C, and most of the supernatant was discarded. The sample was centrifuged again for 5 min at 11,800 rpm at 4°C, and the supernatant was discarded, followed by drying at room temperature. When the RNA precipitate had turned clear, RNase-free water was added until it was completely dissolved, and the concentration and quality of the extracted RNA were determined with a NanoDrop™ 2000/2000C spectrophotometer. cDNA was obtained by reverse transcription, and real-time PCR was performed in two steps.

#### 2.3.9 Statistical methods

Each experiment was repeated three times, and the average value was taken for analysis. SPSS 20.0 software was used for the statistical analysis and to generate plots. The measurement data are expressed as means ± the standard deviation (x ± s). The t-test was used for comparison of means between two groups. ANOVA was used for the comparison of means between multiple groups, and the chi-square test was used for comparison of rates between groups. Values with *P* < 0.05 were considered to indicate a statistically significant difference.

## 3 Results

### 3.1 WB were used to verify decreased expression after KCP knockout

**Figure 1:**
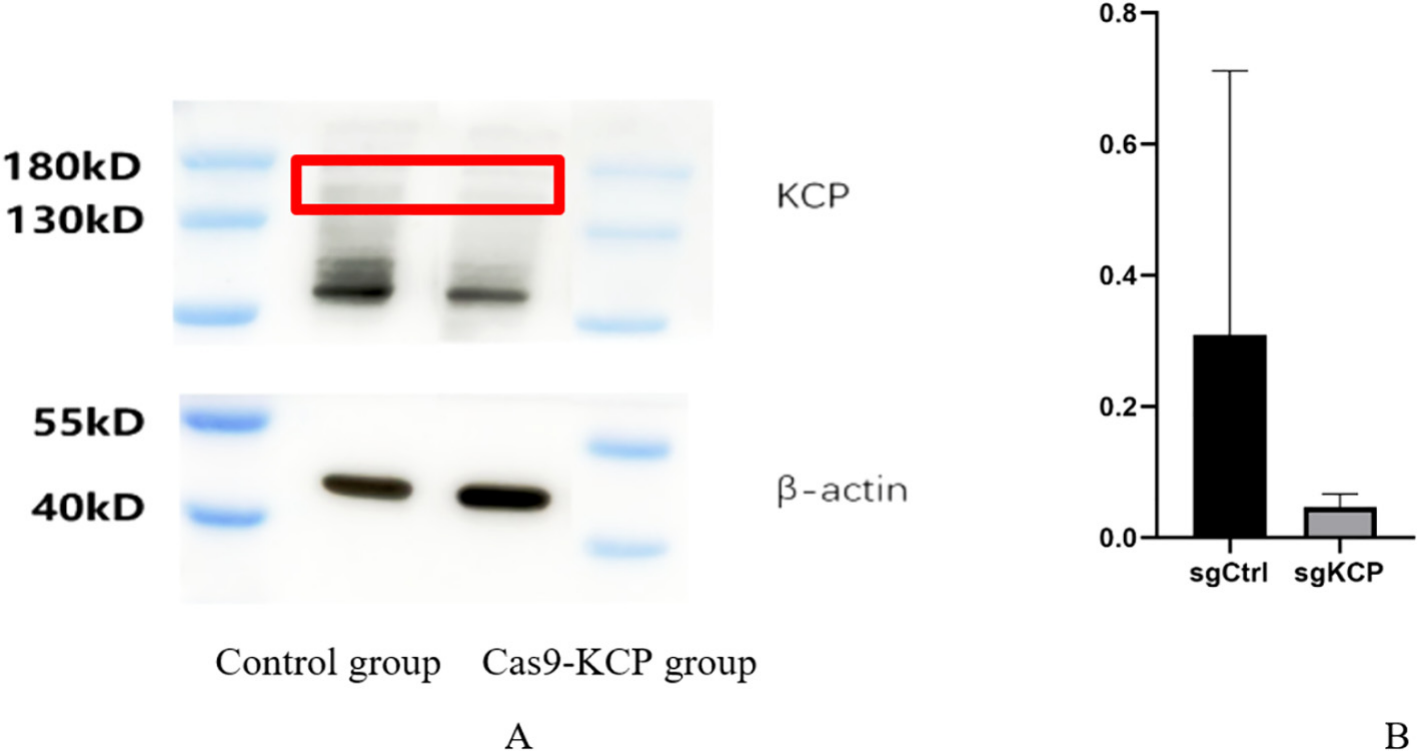
WB were used to verify expression after KCP knockout **(A)**: Western blot demonstrating Cas9-KCP in SiHa cells. A lentivirus for the Cas9-KCP was constructed and co-cultured with SiHa cells. KCP expression was detected in the SiHa cells by western blotting. **(B)**: KCP protein expression was signicantly lower in the Cas9-KCP group than in the control group.

We applied WB for validation of KCP-Cas9 knockout, using β-actin as an internal reference, in the SiHa control group (empty virus) vs SiHa-Cas9 KCP group, and it can be seen that KCP was successfully knocked out (**Figures 1A, B**) The experiment was repeated three times.

### 3.2 Functional studies on the involvement of *KCP* in mediating paclitaxel resistance in cervical cancer cells

#### 3.2.1 Use of the cervical squamous carcinoma cell line SiHa as an *in vitro* model of the influence of *KCP* on paclitaxel resistance

Using CRISPR/Cas9 gene editing, six sgRNAs were designed for the *KCP* gene. Plasmids were constructed, lentiviruses were packaged, and human cervical cancer cell line (SiHa) cells were infected to obtain the *KCP* knockout SiHa strain. We analyzed the ploidy data of each of the six SiHa strains after 120 hours of treatment with 4 ng/ml of paclitaxel or drug solvent (see **Table 1**). The optimal *KCP* knockout target was selected. *KCP* knockout significantly reduced the resistance of cervical squamous cell carcinoma SiHa cells to paclitaxel.

**Table 1:** Assessment of optimal KCP knockout targets using paclitaxel resistance in SiHa cell lines.

Target	Cell ploidy number (120 h)	Sensitization multiplier	Remarks
sgRNA-NC	7.93	1.00	
sgRNA-NC- paclitaxel	2.24		
**KCP-sgRNA-01**	**7.21**	**2.37**	**Optimal target**
**KCP-sgRNA-01- paclitaxel**	**0.86**		
KCP-sgRNA-02	7.18	1.72	
KCP-sgRNA-02- paclitaxel	1.18		
KCP-sgRNA-03	7.34	1.61	
KCP-sgRNA-03- paclitaxel	1.29		
KCP-sgRNA-04	7.49	1.47	
KCP-sgRNA-04- paclitaxel	1.44		
KCP-sgRNA-05	7.21	1.34	
KCP-sgRNA-05- paclitaxel	1.52		
KCP-sgRNA-06	7.15	1.15	
KCP-sgRNA-06- paclitaxel	1.75		

Bold values indicates optimal KCP knockout targets.

#### 3.2.2 Effects on colony formation

**Figure 2:**
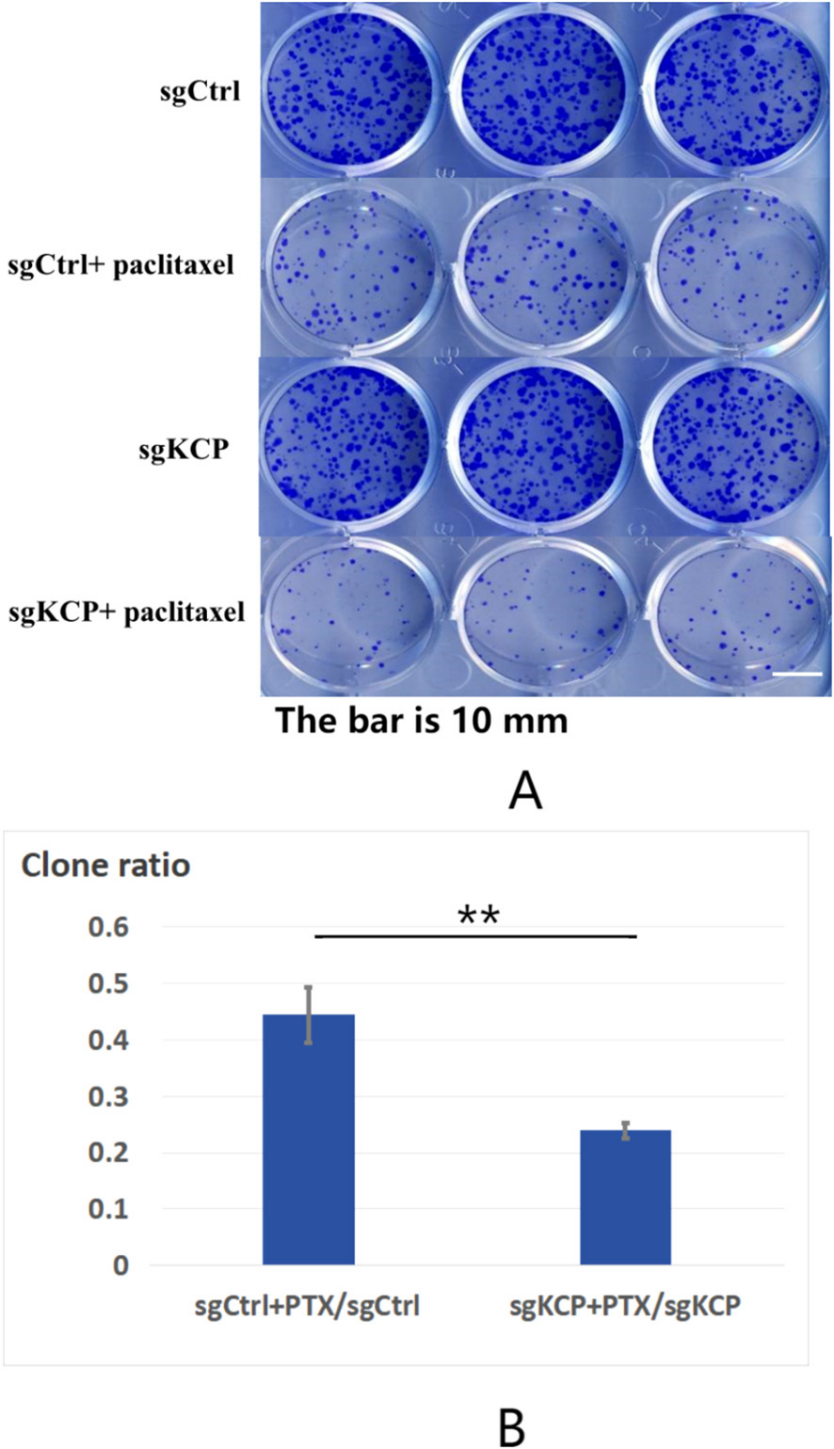
Effect of *KCP* knockout on SiHa cell colony formation, the experiment was repeated three times **(A)**: The effect of *KCP* knockout on cervical squamous cell carcinoma SiHa colony formation. **(B)**: The inhibition of SiHa cell colony formation after *KCP* knockdown increased the sensitivity to paclitaxel. sgCtrl: sgRNA-negative control virus-infected SiHa cells; sgCtrl+paclitaxel: paclitaxel-treated cells infected with sgRNA-negative control virus sg*KCP*: cells infected with sgRNA virus specific to the *KCP* gene; sg*KCP*+paclitaxel: paclitaxel-treated cells infected with the sgRNA virus specific to the *KCP* gene.

The effect of *KCP* knockout on cervical squamous cell carcinoma SiHa colony formation was assayed. The cells were cultured for 9 days, and the number of cell colonies was then counted. The study group was incubated with or without paclitaxel (2ng/ml) on the fourth day. The results show that the number of SiHa cell colonies was as follows: 245 ± 11 in the sgCtrl group, 108 ± 8 in the sgCtrl+paclitaxel group, 233 ± 11 in the sg*KCP* group, and 56 ± 1 in the sg*KCP* + paclitaxel group. No statistically significant difference was observed between the sgCtrl and sg*KCP* groups (*p*=0.2755) and between the sgCtrl+paclitaxel and sg*KCP*+paclitaxel groups (*p*=0.0004). Compared to the sgCtrl+paclitaxel group, the sg*KCP*+paclitaxel group exhibited a significantly less potent SiHa cell colony formation ability, and the inhibition of SiHa cell colony formation after *KCP* knockdown increased the sensitivity to paclitaxel (**Figure 2**). The experiment was repeated three times.

#### 3.2.3 Effects on cell proliferation

**Figure 3:**
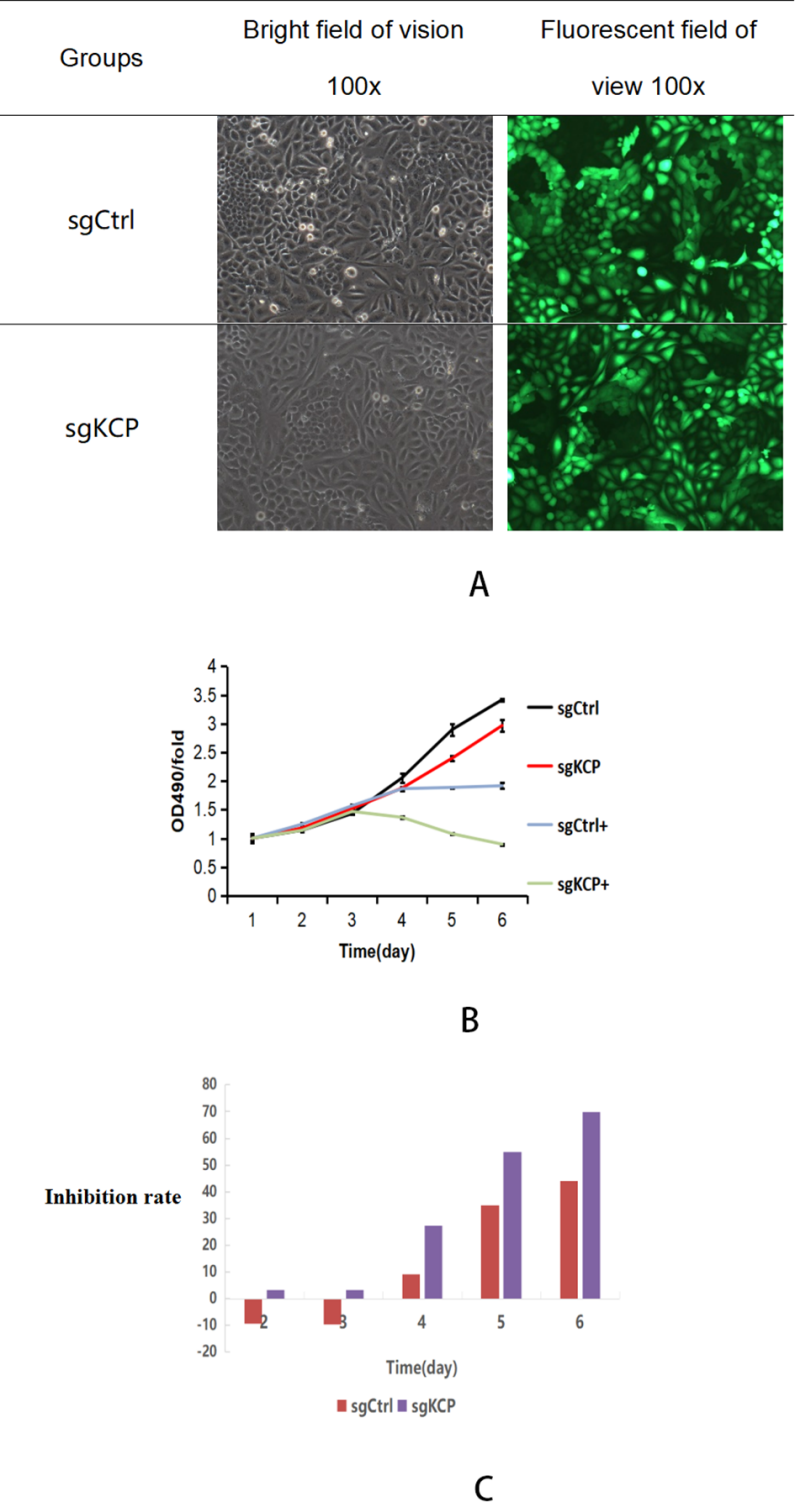
MTT assay of SiHa cell proliferation after infection with sg*KCP*, the experiment was repeated three times **(A)**: Comparison of changes in the light absorption measured at a wavelength of 490 nm using a microplate reader after treatment of sgRNA lentivirus-infected SiHa cells with MTT for four hours following incubation with paclitaxel. OD490/fold was the OD490 increase relative to day 1 for each experimental group from day 1 to day 6, representing the proliferation increase for each day. **(B)**: Comparison of the inhibition of each cell group after the addition of paclitaxel, inhibition rate: [1-OD490/fold(sg*KCP*/sg*KCP* + paclitaxel)/OD490/fold(sgCtrl/sgCtrl + paclitaxel)] × 100. **(C)**:The inhibition rate was significantly higher for the sg*KCP* group compared to that for the sgCtrl group after the addition of paclitaxel.

To investigate the effect of *KCP* knockdown on cervical squamous carcinoma SiHa cell proliferation, we conducted experiments on SiHa cells infected with lentiviruses containing the target gene. After 72 hours of infection by the control or target lentivirus, observation of the cells by fluorescence microscopy showed that the infection efficiency was over 70% (**Figure 3A**). sgCtrl and sg*KCP* groups were incubated with or without paclitaxel (4ng/ml) for 6 days and treated with MTT for four hours. The changes in the light absorption at 490 nm were recorded separately for each group using a microplate reader. The OD490 reflects the number of viable cells. The results revealed a statistically significant difference between the sgCtrl and sg*KCP* groups (*p*=0.00219) and between the sgCtrl+paclitaxel and sg*KCP*+paclitaxel groups (*p*=0.000005). Compared to the sgCtrl+paclitaxel group, the cell proliferation in the sg*KCP*+paclitaxel group was significantly reduced, indicating that the cells in this group were more sensitive to paclitaxel. The experiment was repeated three times, the results are shown in **Figure 3B**.

The inhibition rate of each cell group was compared after the addition of paclitaxel. The inhibition rate was significantly higher for the sg*KCP* group compared to that for the sgCtrl group, and the sensitivity to paclitaxel was increased after *KCP* knockout. The results are shown in **Figure 3C**.

#### 3.2.4 Unaltered apoptosis rate of the cervical squamous cell carcinoma cell line SiHa after treatment with paclitaxel

**Figure 4:**
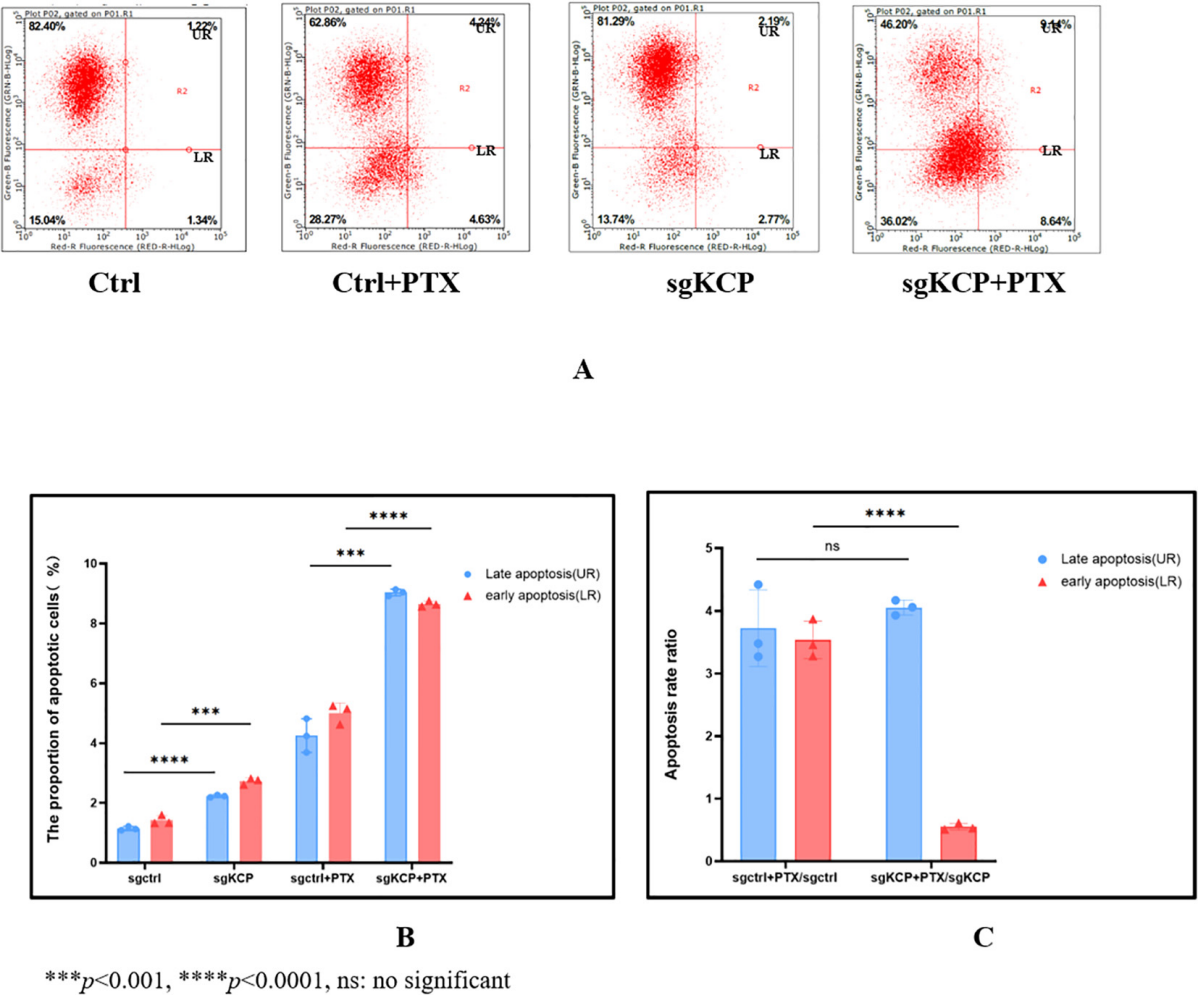
Effect of KCP knockout on SiHa cell apoptosis after treatment with paclitaxel, the experiment was repeated three times **(A)**: Apoptosis and necrosis rates, UR: upper right (Late apoptosis), LR: lower right (Early apoptosis) **(B)**: The proportion of early vs. late apoptosis in different group, the differences of early and late apoptosis between sgCtrl vs. sgKCP and sgCtrl+PTX vs. sgKCP+PTX were statistically significant. **(C)**:The total apoptosis rate ratios were as follows: sgCtrl+paclitaxel/sgCtrl=3.6 and sg*KCP*+paclitaxel/sg*KCP*=3.57 (No significant, ns), the late apoptosis rate ratio of *KCP* knockout SiHa cells was not affected by paclitaxel treatment compared to the control group (ns), but the early apoptosis rate ratio of *KCP* knockout SiHa cells was lower compared to the control group (*p*<0.0001).

The effect of *KCP* knockdown and control lentivirus on apoptosis in the cervical squamous carcinoma SiHa cell line was examined with and without paclitaxel (4ng/ml). No significant difference was observed between the apoptosis ratios of the groups. The results of the study revealed that the total apoptosis rate ratios were as follows: sgCtrl+paclitaxel/sgCtrl=3.6 and sg*KCP*+paclitaxel/sg*KCP*=3.57 (No significant, ns), the late apoptosis rate ratio of *KCP* knockout SiHa cells was not affected by paclitaxel treatment compared to the control group (ns), but the early apoptosis rate ratio of *KCP* knockout SiHa cells was lower compared to the control group (*p*<0.0001). The experiment was repeated three times, the results are shown in **Figure 4**.

### 3.3 Human gene chip detection and IPA bioinformatics analysis

The total RNA of SiHa cells from the sg*KCP* and control lentivirus groups was extracted for gene chip assay. A total of 491 differentially expressed genes were screened by IPA bioinformatics analysis to examine if other factors may be related to the effects of *KCP* gene knockout. We also studied the mechanism of action underlying the effect of *KCP* on tumor cell proliferation. The IPA bioinformatics analysis included examination of regulatory effects, disease and function, classical pathways, upstream regulatory factors, and interaction networks. Ultimately, 30 possible *KCP* downstream genes were screened and validated using qPCR.

#### 3.3.1 Classical pathways based on IPA bioinformatics analysis

**Figure 5:**
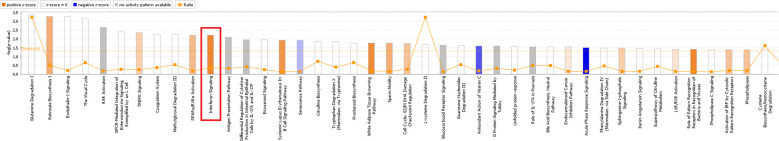
Classical pathway enrichment analysis. The abscissa indicates the name of the pathway, and the ordinate represents the enrichment significance level. The orange label indicates activation of the pathway. The blue label indicates inhibition of the pathway, and the depth of the orange and blue colors represents the degree of activation or inhibition. The ratio represents the number of differential genes in the signaling pathway to the number of all genes included in the pathway (The main genes are marked in red).

**Figure 6:**
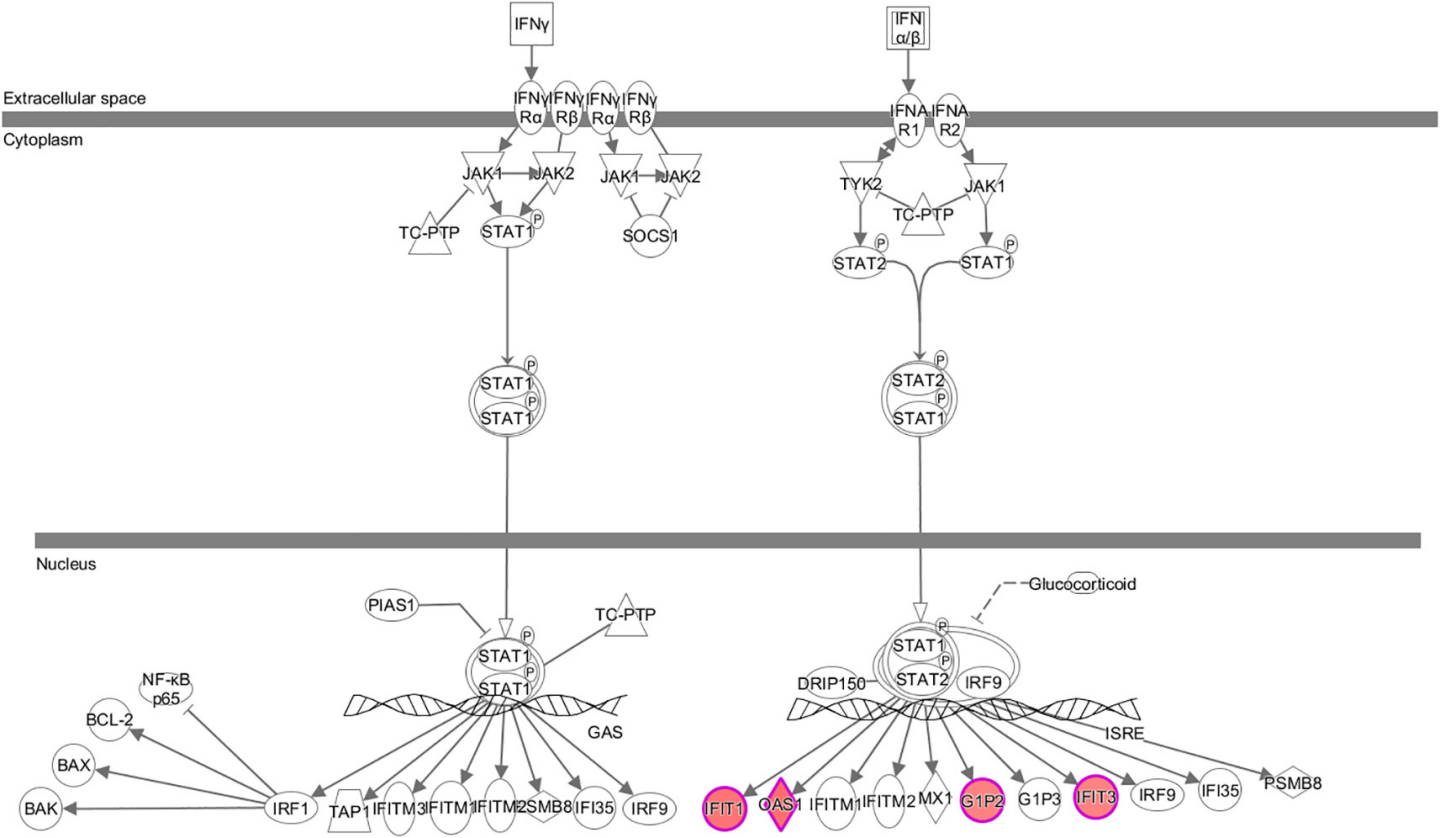
Expression trends of each molecule associated with the interferon signaling pathway based on gene chip analysis The interferon signaling pathway was significantly activated. Red color indicates that the gene was significantly upregulated, and green color indicates that the gene was significantly downregulated in the experimental results (The main genes are marked in red).

Significant enrichment of differentially expressed genes in the classical pathways showed that 11 signaling pathways were significantly activated and three pathways were inhibited. The significantly activated signaling pathways included: the interferon (Z-score=2.0), role of pattern recognition receptors in recognition of bacteria and viruses (Z-score=2.0), corticotropin-releasing hormone g (Z-score=2.0), protein kinase A (Z-score=2.111), cardiac hypertrophy (Z-score=2.111), GPCR-mediated nutrient sensing in enteroendocrine cells (Z-score=2.236), P2Y purinergic receptor (Z-score=2.236), AMPK pathway (Z-score=2.236), dopamine-DARPP32 feedback in cAMP (Z-score=2.236), GNRH s (Z-score=2.236), and cardiac hypertrophy signaling pathways (Z-score=2.449). The significantly inhibited signaling pathways included the acute phase response (Z-score=-2.0), acute myeloid leukemia (Z-score=-2.0), and PI3K/AKT signaling pathways (Z-score=-2.0). Furthermore, the interferon signaling pathway was closely related to the tumor pathway (see **Figure 5**). The human gene microarray analysis showed that four differentially expressed genes, namely, *IFIT1, OAS1, G1P2*, and *IFIT3*, were important factors involved in mediating the interferon signaling pathway, suggesting that *KCP* may be involved in the interferon signaling pathway (**Figure 6**).

#### 3.3.2 Upstream regulatory factors based on IPA bioinformatics analysis

**Figure 7:**
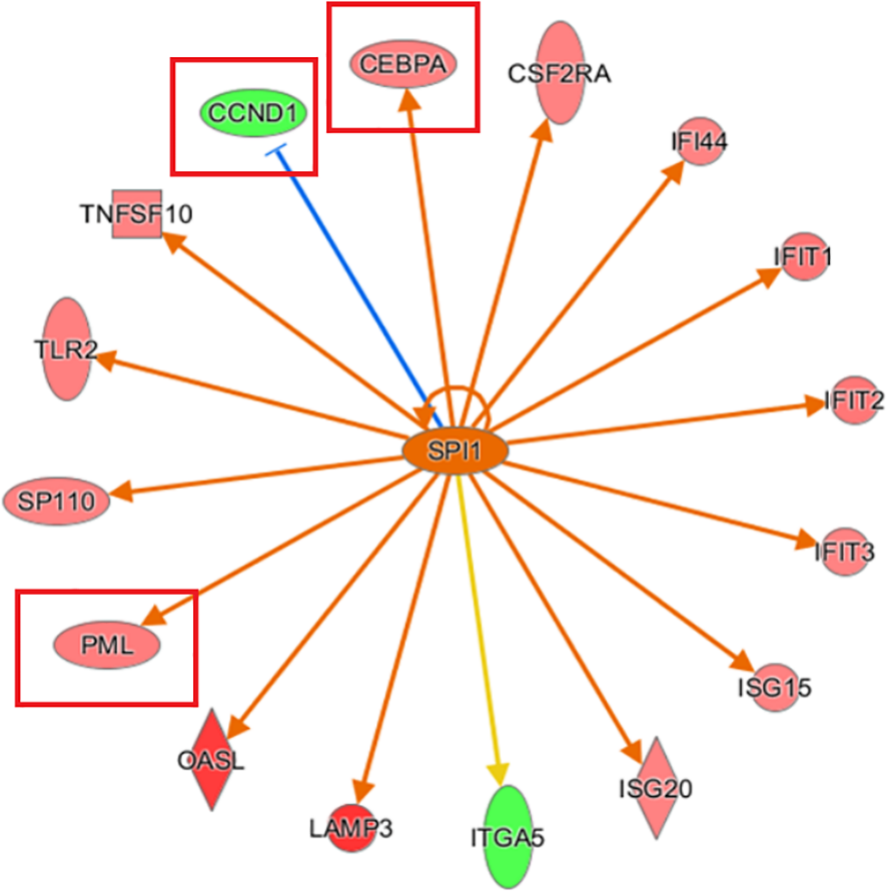
Network diagram of upstream regulatory factors based on IPA analysis Interaction relationships between the upstream regulatory factors based on IPA analysis, and directly related and co-existing downstream molecules in the dataset. The orange line indicates consistent activation of the upstream regulators and the gene. The blue line indicates consistent inhibition of the upstream regulators and the gene. The yellow line indicates inconsistent expression trends between the upstream regulators and the gene, and the grey line indicates the lack of predictive information related to the expression state in the dataset (The main genes are marked in red).

Analysis of the upstream regulatory factors revealed that SPI1 was strongly activated, and 15 consistently activated genes were identified. SPI1-inhibited *CCND1* activated both *PML* and *CEBPA* simultaneously, which is consistent with the observation of *CCND1*, *PML*, and *CEBPA* expression following *KCP* knockout (**Figure 7**).

#### 3.3.3 Diseases and functions examination by IPA bioinformatics analysis

**Figure 8:**
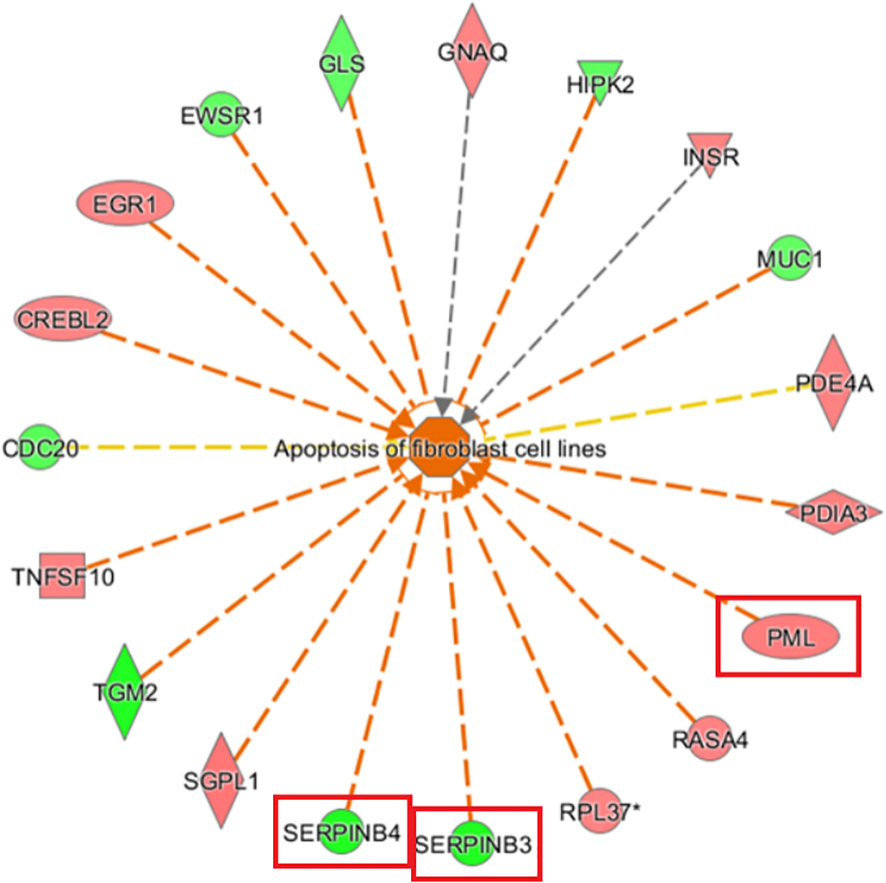
Gene disease or function network map based on IPA analysis The orange line indicates that changes in gene expression levels had an activating effect on this function. The blue line indicates that the changes in gene expression levels had an inhibiting effect on this function, and the yellow line indicates that changes in gene expression levels affected this gene function, which is inconsistent with existing literature reports. The grey line indicates that the regulatory relationship was unknown (The main genes are marked in red).

Examination of diseases and functions based on IPA analysis showed significant activation of apoptosis of fibroblast cell lines (Z-score= 3.090) and cell death of connective tissue cells (Z-score= 2.998) and significant inhibition of replication of vesicular stomatitis virus (Z-score=-2.834) and abdominal carcinoma (Z-score=-2.337). *PML* and *SERPINB3/4* (squamous cell carcinoma antigen 1, SCCA1) were key genes associated with the most significant activation of apoptosis of fibroblast cell lines (**Figure 8**).

#### 3.3.4 Analysis of regulatory effects by IPA bioinformatics

**Figure 9:**
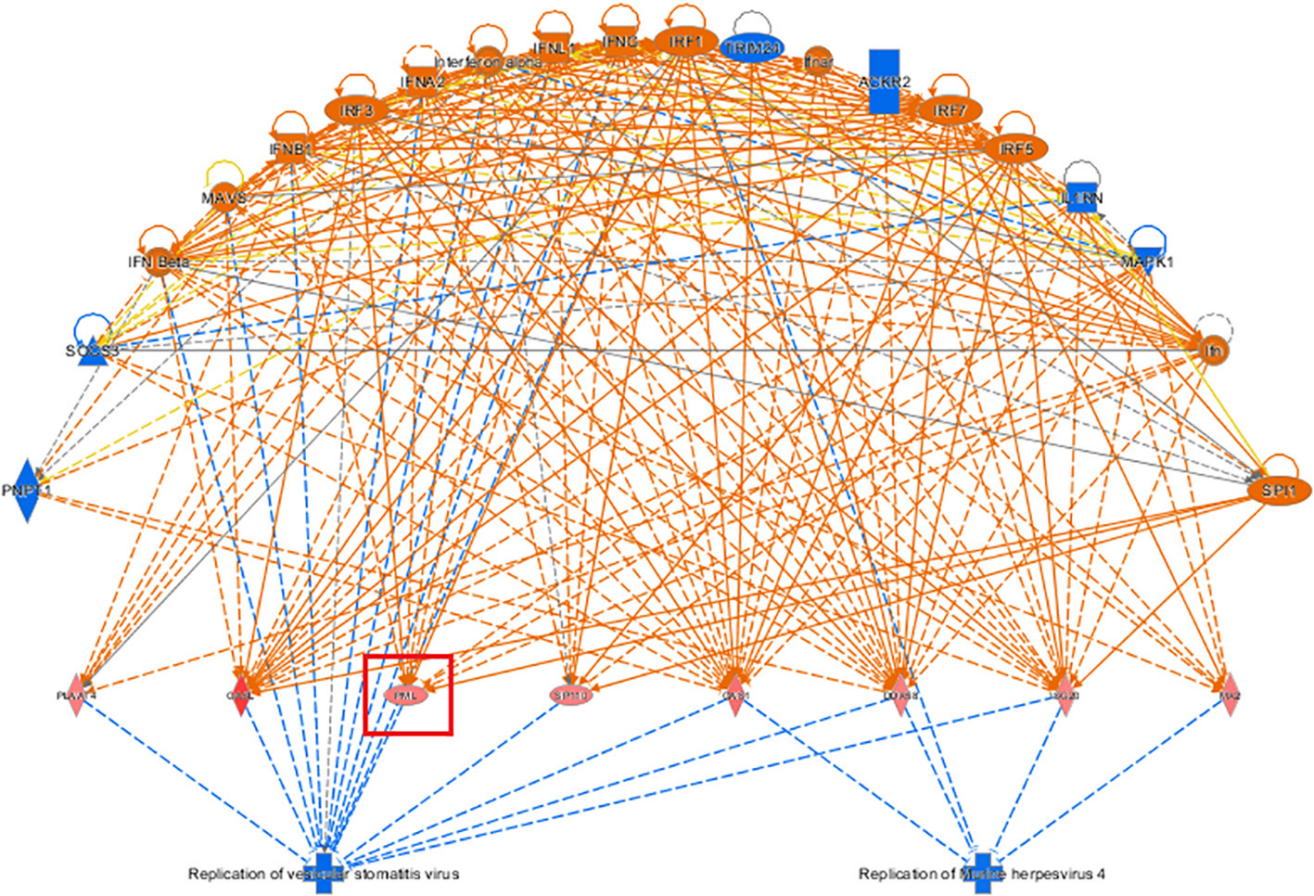
Network diagram of regulatory effects Showcased the interaction between genes, regulators, and functions in the dataset. The top-ranked regulatory network in terms of regulatory effects based on the IPA-based analysis showed that it may be due to the inhibitory effects of differentially expressed genes on the regulators, replication of murine herpesvirus 4 and replication of vesicular stomatitis virus, thereby affecting the gene function of *KCP* (The main genes are marked in red).

The top-ranked regulatory network in terms of regulatory effects according to the IPA-based analysis showed that it may involve the inhibitory effects of the differentially expressed genes *ACKR2, Ifn, IFN Beta, IFNA2, Ifnar, IFNB1, IFNG, IFNl1, Il1RN*, Interferon alpha*, IRF1, IRF3, IRF5, IRF7, MAPK1 MAVS, PNPT1, SOCS3, SPI1, TRIM24* through *DDX58, ISG20, MX2, OAS1, OASl, PlAAT4, PML*, and *SP110* on the regulators, replication of murine herpesvirus 4 and replication of vesicular stomatitis virus, thereby affecting the gene function of *KCP* (see **Figure 9**).

**Figure 10:**
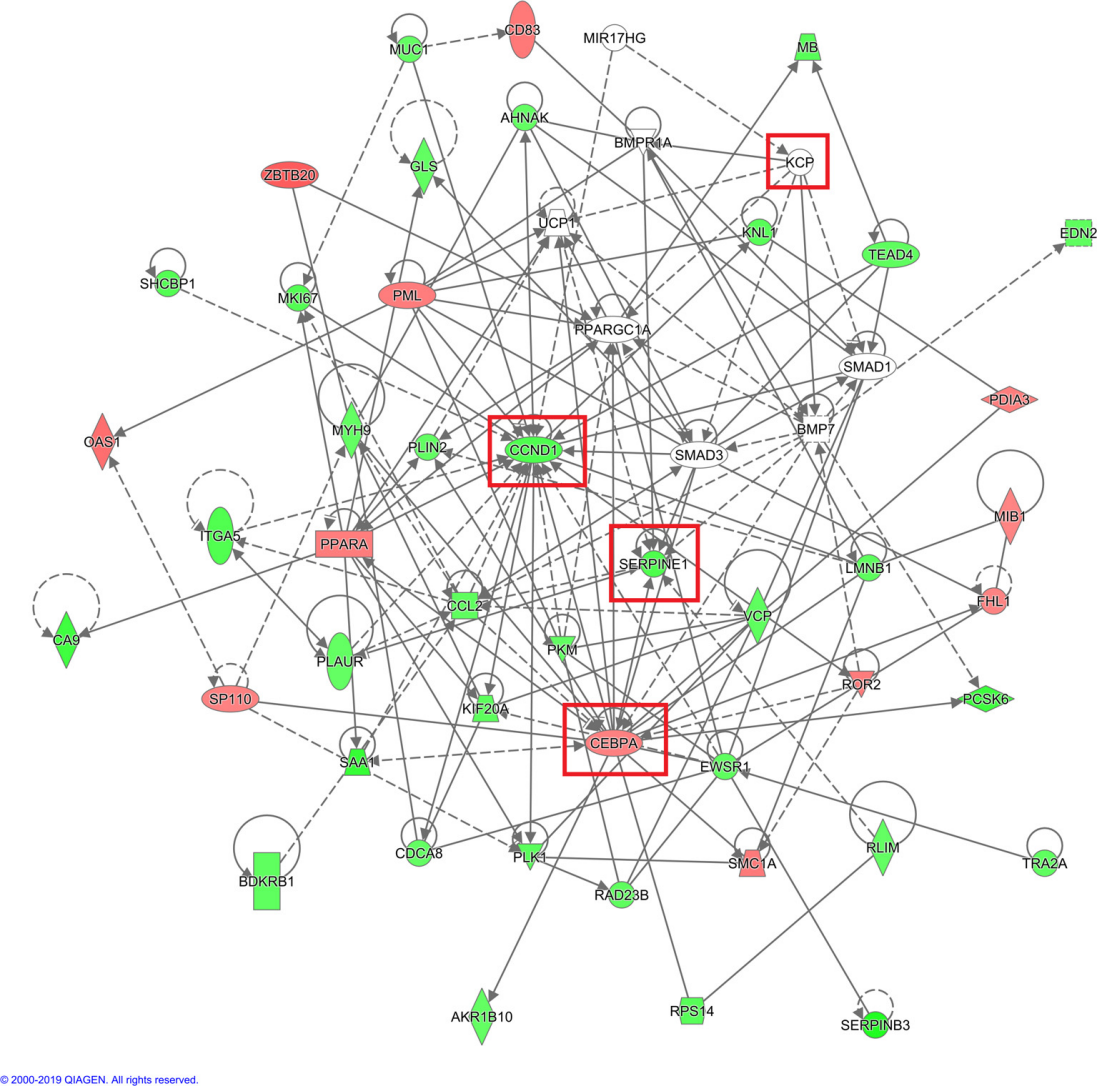
IPA analysis of gene interaction networks. Red color indicates that increased measurement, green color indicates that decreased measurement, darker color prompt more extreme increased measurement in dataset (The main genes are marked in red). Net work shapes: □ Cytokine, ◊ Enzyme, ▯ G-protein coupled receptor, ▽ Kinase, ▭ Ligand-dependent nuclear receptor, 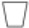
microRNA, 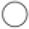
other, 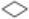
Peptidase, 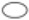
Transcription regulator, 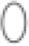
Transmembrane receptor, 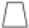
Transporter.

In the IPA analysis of differentially expressed genes in the KO vs. NC groups, genes related to tumor cell proliferation and apoptosis were identified in the disease function analysis results. Genes that inhibit tumor cell proliferation or that promote tumor cell apoptosis were selected, and KCP target genes were added and the gene relationship network diagram was drawn. The comprehensive results of the final IPA analysis indicate that KCP may be indirectly involved in the mechanism underlying paclitaxel resistance via the association of PML and CCND1 genes, thereby affecting the proliferation of SiHa cells (**Figures 10**).

### 3.4 Validation of the mechanism downstream of *KCP* knockout

**Figure 11:**
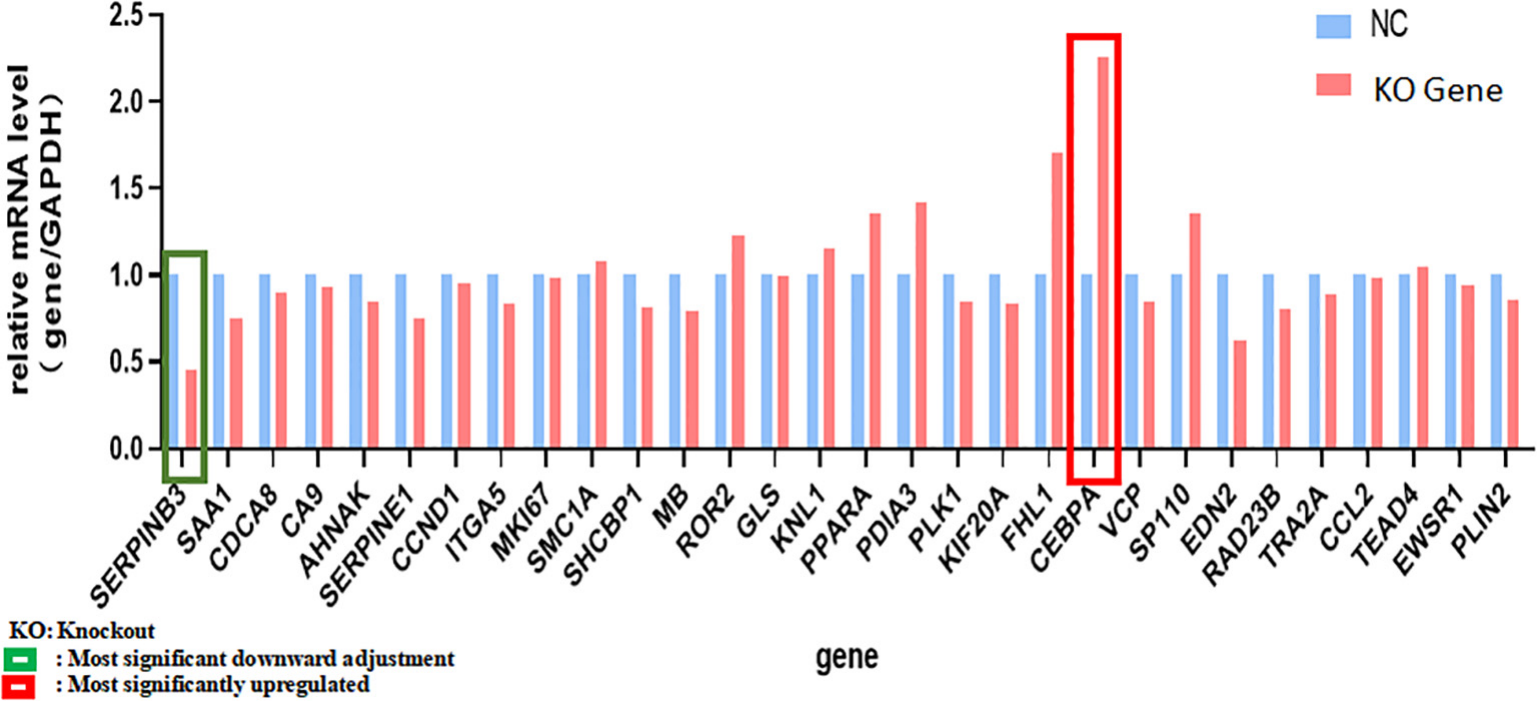
qPCR analysis verified genes in **Table 2** qPCR was used to verified genes in **Table 2**. Green frame is the most significant downward adjustment, red frame is the most significantly upregulated (The main genes are marked in red).

The differentially expressed genes screened based on the human gene chip were analyzed by IPA bioinformatics. A total of 52 genes associated with tumor cell proliferation were found by the disease function analysis. A total of 30 genes with the most significant differences were screened by qPCR for validation. Of these, *SERPINB3* and *CEBPA* exhibited significantly lower and higher expression in KCP knockout group, respectively, compared to their expression in the control group. *PML* was not ranked among the top 30 genes due to its lesser difference, and hence, no follow-up validation was performed. The qPCR analysis verified that *CCND1* had a slightly reduced expression. Therefore, we hypothesize that *SERPINB3* and *CEBPA* may be forward and reverse downstream genes of *KCP*, respectively (see **Table 2**; **Figure 11**). *CEBPA* is a key factor of the acute myeloid leukemia signaling pathways, which may be significantly inhibited by *KCP*, as observed by the traditional IPA bioinformatics pathway analysis. *SERPINB3* is the key gene that most significantly activates apoptosis of fibroblast cell lines, as shown by the IPA bioinformatics disease and functional analysis. As *KCP* is located in the extracellular matrix, further validation is required to elucidate its involvement in the transcriptional function of genes related to *CCND1*.

**Table 2:** Validation of the downstream gene mechanism of KCP knockout.

S/N	Name	Upstream primer sequence	Downstream primer sequence	Expression abundance in NC group
Control	GAPDH	TGACTTCAACAGCGACACCCA	CACCCTGTTGCTGTAGCCAAA	Internal control
1	SERPINB3	GATCGCCAACAAGCTCTTCGG	ATTCGTTTGACTTTCCACCC	0.45 (most significant downward adjustment)
2	SAA1	TTCCTTGGCGAGGCTTTTGA	TCCCCTTTTGGCAGCATCAT	0.76
3	CDCA8	GCAGGAGAGCGGATTTACAAC	CTGGGCAATACTGTGCCTCTG	0.90
4	CA9	GGATCTACCTACTGTTGAGGCT	CATAGCGCCAATGACTCTGGT	0.93
5	AHNAK	TGCCACCATCTACTTTGAC	GTTCTGGTCTTTGCATTCC	0.85
6	SERPINE1	GAGAACCTGGGAATGACCGAC	CATGCGGGCTGAGACTATGAC	0.75
7	CCND1	CCTCACACGCTTCCTCTC	CCTCCTCTTCCTCCTCCTC	0.96
8	ITGA5	GGCTTCAACTTAGACGCGGAG	TGGCTGGTATTAGCCTTGGGT	0.84
9	MKI67	GGAACAGCCTCAACCATCAG	CCACTCTTTCTCCCTCCTCTC	0.98
10	SMC1A	AGCGAAAGGCAGAGATAATGG	GGTAGTCAAGAGGCAAGAAGG	1.08
11	SHCBP1	AGGTTATGGTGTTGTCTTGGTG	TCGACTTGCTCAGAAGGGTCA	0.81
12	MB	CACCAAGCACAAGATCCCC	GCATCAGCACCAAAGTCCC	0.80
13	ROR2	CCTTTAGGACCCCTTGATG	CACCTTGCAGTGCAGAATT	1.22
14	GlS	GAAAAGAGCCGAGTGGAC	AACCATGTCTGTGCCTTC	1.00
15	KNl1	TCTCAACCGACCAACCTCCAT	ACGTCTGAGTTACTGTGGAAAT	1.15
16	PPARA	CCTCCTCGGTGACTTATCCTG	CGACAATACTGGCATTTGTTTC	1.35
17	PDIA3	CTTGAGGGATAACTACCGATTT	TTAGTGAGATGTGAAGGACGAA	1.42
18	PlK1	AGGCAAGAGGAGGCTGAG	GGATGAGGCGTGTTGAGTC	0.84
19	KIF20A	TGCTGTCCGATGACGATGTC	AGGTTCTTGCGTACCACAGAC	0.84
20	FHL1	GACAATCCTGGCACGACTAC	TCCTGGTGGAAAACAAAGC	1.71
21	CEBPA	CCAGAAAGCTAGGTCGTGGGT	TGGACTGATCGTGCTTCGTGT	2.25 (most significantly upregulated)
22	VCP	TCTGATGATACTTGTTCTGATGAG	ATGGCTGGATGCTGATGAC	0.84
23	SP110	AGAAGACGCCTAGTACACC	TGAGTCACCTTATCCACCA	1.36
24	EDN2	GTCCTCATCTCATGCCCAAG	CCGTAAGGAGCTGTCTGTTC	0.63
25	RAD23B	TTCCACACCTGCATCCATCAC	TCAGTTGCTGTTGGGCTAGTA	0.81
26	TRA2A	TCTCGTAGTCCATCAAGGGTT	TGCTGCCAGTATGTCTTCTCC	0.88
27	CCl2	CAGCCAGATGCAATCAATGCC	TGGAATCCTGAACCCACTTCT	0.98
28	TEAD4	GGACACTACTCTTACCGCATCC	TCAAAGACATAGGCAATGCACA	1.05
29	EWSR1	ATGGCGTCCACGGATTACAG	GTGCATATCCTTGAGTGGGCT	0.95
30	PlIN2	TTGCAGTTGCCAATACCTATGC	CCAGTCACAGTAGTCGTCACA	0.86

## 4 Discussion

Cervical cancer is a serious health risk for women. Paclitaxel-based chemotherapy is pivotal in the treatment of advanced and recurrent cervical cancer as the primary treatment. Currently, paclitaxel-based chemotherapy regimens have been included in the guidelines of the National Comprehensive Cancer Network (NCCN) (10), the International Federation of Gynecology and Obstetrics and Gynecology (FIGO), and the European Society for Medical Oncology (ESMO) for the standard treatment of intermediate to advanced cervical cancer. However, in patients treated with paclitaxel as first-line chemotherapy, the drug resistance rates associated with intermediate to advanced squamous cervical cancers range from 20% to 44% (11). Paclitaxel resistance in patients with cervical squamous cell carcinoma leads to a progression of the disease, thus affecting the prognosis of patients and reducing the five-year survival rate by 20% to 30%. Studies in the USA and Japan have reported (12, 13) that 20% of patients with locally advanced squamous cell cervical cancer are resistant to paclitaxel-based neoadjuvant chemotherapy, resulting in treatment failure. Paclitaxel resistance is a major cause of treatment failure in patients with cervical squamous cell carcinoma. Currently, no specific markers or targets for drug resistance in cervical cancer are known.

In 2002, Abreu’s research group discovered that *KCP* could affect the progression of certain chronic and metabolic diseases via the regulation of extracellular signaling. *KCP* comprises a cysteine structural domain with a secretory function and is generally highly expressed in the germ and kidney structures during embryonic development, but there is little expression in normal adult tissues. Current research on *KCP* has focused on chronic liver and kidney fibrosis and metabolic diseases (14, 15), suggesting that it can affect cell proliferation, migration, and adhesion (11). Recent studies examining the pathogenic mechanisms underlying cervical cancer have focused on the Wnt, Notch, BMP, and Jak-Stat3 signaling pathways (16). The results show that the most common regulatory pathway involves binding of *KCP* to TGF-β receptors, thereby inhibiting their signaling and promoting receptor-ligand interactions in a paracrine manner. Furthermore, enhancement of the BMP signaling pathway has also been observed. The binding of *KCP* to BMP7 enhances the binding to type I receptors, which in turn increases Smad1-dependent transcription and phosphorylated Smad1 (P-Smad1) levels (17–19). TGF-β and BMP regulatory pathways play important roles in the proliferation and invasion of various malignant tumors.

In this study, we successfully generated a *KCP* knockout cervical squamous cell carcinoma *in vitro* model using CRISPR/Cas9 gene editing technology and preliminarily confirmed the regulatory function of *KCP* in SiHa cell line. Further testing confirmed the involvement of *KCP* in paclitaxel resistance of cervical cancer cells by examination of cell colony formation and proliferation. The study showed that *KCP* knockout significantly inhibited colony formation and proliferation of cervical cancer cells in the paclitaxel group, thereby increasing the sensitivity to paclitaxel. The differentially expressed genes after the *KCP* knockout were analyzed by human whole genome microarray and IPA bioinformatics analyses, and the downstream genes and mechanisms were explored.

The IPA bioinformatics analysis revealed that the most important classical pathway for *KCP* activation was the interferon signaling pathway, and the downstream factors included the JAK-STAT signaling pathway as well as BAX, BAK, BCl-2, and other possible key factors That is partially consistent with the recent report mentioned in the previous paragraph (20).The analysis of the IPA regulatory network showed that the aforementioned regulation may be associated with the regulatory network of the replication of the murine herpesvirus 4 and the replication of the vesicular stomatitis virus. This indicated that *KCP* may be indirectly involved in cell proliferation via *PML* and *CCND1.* further experiments are required to confirm the mechanism of CCND1 suppression and *PML* activation by observing the activation of SPI1 after *KCP* knockout. According to the IPA analysis of differentially expressed genes, qPCR was used to verify KCP could also act positively on the downstream gene of SERPINB3 and negatively on the downstream gene of CEBPA to affect the resistance of cervical carcinoma to paclitaxel. It has been reported that CEBPA is positively related to chemoresistance and that CEBPA overexpression can significantly enhance chemoresistance in oral squamous cell carcinoma (OSCC) (20, 21). The forward downstream gene, *SERPINB3*, as validated by qPCR, may be the key factor that significantly activates the disease and functions during the apoptosis of fibroblast cell lines. The endogenous lysosomal cysteine protease inhibitor *SERPINB3* (SCCA1) is elevated in patients with cervical cancer and other malignancies. Cervical cancer cells lacking *SERPINB3* are more sensitive to ionizing radiation (IR), suggesting that this protease inhibitor plays a role in the therapeutic response. t has been reported that SERPINB3 confers resistance to drug-induced apoptosis by inhibiting lysosomal cathepsin proteases in cancer cells (22). SERPINB3 enhances the survival ability of tumor cells by regulating cytokine activity and signaling pathways related to tumor development (23). Wang et al. demonstrated that SERPINB3-deficient cells had enhanced sensitivity to IR-induced cell death. Knockout of *SERPINB3* sensitizes cells to a greater extent than cisplatin (24, 25). However, the reverse downstream gene, *CEBPA*, is a key factor involved in the significant inhibition of the disease and function-related signaling pathways in acute myeloid leukemia signaling. CEBPA participates in chemoresistance through METTL3/METTL14/BHLHB9 *in vivo*, which accelerated the tumor growth (26). Therefore, the involvement of *KCP* in related diseases and functions through the downstream factors SERPINB3 and CEBPA should also be studied in the future.

PTX resistance is the main cause of treatment failure in cervical cancer. Therefore, developing a strategy to overcome the PTX resistance has become the most critical clinical subject for gynecologic oncologists. Considering the close relationship between KCP and paclitaxel resistance, KCP can be used as a candidate gene for paclitaxel resistance markers and therapeutic targets in cervical cancer in the future.

## 5 Summary

The incidence and death rates of cervical cancer still rank it as the foremost malignant tumor of the female reproductive system in China. The process of onset and development is very complex, with involvement of multiple genes, molecular proteins, biological factors, and signaling pathways in its regulation. Chemotherapy resistance is a common cause of recurrence. In recent years, even though a degree of progress has been made in gene therapy and molecular targeted therapy for cervical cancer, it is still crucial to explore the molecular mechanism of chemotherapy resistance in cervical cancer in depth, to find effective therapeutic targets, to design new innovative drugs developed independently in China, to reduce the incidence of chemotherapy resistance, to prolong the OS and PFS of patients with tumor, and to improve their quality of life. This study suggests that *KCP*, which is an emerging protein, plays an important role in the proliferation of tumor cells, and its related mechanism has also been partially explored. We aim to explore more medical evidence, which is expected to provide a new direction to overcome the drug resistance of cervical cancer.

## 6 Limitations

This paper has some limitations:

1. Only one cell line of KCP knockout was established in this research. We discuss the paclitaxel resistance gene in cervical squamous cell carcinoma. SiHa is a human papilloma virus-related cervical squamous cell carcinoma line, resistant to cisplatin, we use this cell line to preliminarily explore the function of KCP in cervical squamous cell carcinoma. In future research, we will use various cell lines such as SiHa, Caski, HeLa, etc. to explore the mechanism of KCP’s involvement in paclitaxel resistance in cervical cancer.

2. A total of 30 genes with the most significant differences were screened for qPCR validation. However, it is not been confirmed that these differentially expressed genes are related to paclitaxel resistance.

3. The study of molecular mechanisms is mainly based on speculation of bioinformatics results and lacks cell and animal experimental confirmation.

## 7 Future perspective

1. We will conduct experiments on sensitive and resistant to paclitaxel squamous cervical cancer tumor tissues in the future to confirm the relationship of these genes with paclitaxel resistance.
2. The role of KCP in infiltration, invasion and metastasis in cervical cancer was not evaluated in the present study, we will conduct these experiments in the future.
3. We have preliminarily validated the differential genes that may be involved in paclitaxel resistance in cervical cancer screening, and our subsequent research will arrange experiments to clarify the downstream related genes of KCP.
4. We plan to perform animal experiments to supplement for the verification of KCP and related genes in the future.

## Data Availability

The datasets presented in this study can be found in online repositories. The names of the repository/repositories and accession number(s) can be found in the article/supplementary material.
